# Effect of Low-Dose Radiotherapy on the Circulating Levels of Paraoxonase-1-Related Variables and Markers of Inflammation in Patients with COVID-19 Pneumonia

**DOI:** 10.3390/antiox11061184

**Published:** 2022-06-16

**Authors:** Elisabet Rodríguez-Tomàs, Johana C. Acosta, Laura Torres-Royo, Gabriel De Febrer, Gerard Baiges-Gaya, Helena Castañé, Andrea Jiménez, Carlos Vasco, Pablo Araguas, Junior Gómez, Bárbara Malave, Miguel Árquez, David Calderón, Berta Piqué, Manel Algara, Ángel Montero, Josep M. Simó, Xavier Gabaldó-Barrios, Sebastià Sabater, Jordi Camps, Jorge Joven, Meritxell Arenas

**Affiliations:** 1 Department of Radiation Oncology, Hospital Universitari Sant Joan de Reus, Institut d’Investigació Sanitària Pere Virgili, Universitat Rovira i Virgili, 43204 Tarragona, Spain; elisabet.rodriguez@urv.cat (E.R.-T.); johanac.acostaa@gmail.com (J.C.A.); lauratroyo@gmail.com (L.T.-R.); pablo.araguasmora@gmail.com (P.A.); junioranderson.gomez@gmail.com (J.G.); barbaraantonia.malave@salutsantjoan.cat (B.M.); miguelarquez@gmail.com (M.Á.); victordavid.calderon@salutsantjoan.cat (D.C.); berta.pique@estudiants.urv.cat (B.P.); 2 Unitat de Recerca Biomèdica, Institut d’Investigació Sanitària Pere Virgili, Universitat Rovira i Virgili, 43003 Tarragona, Spain; gerard.baiges@estudiants.urv.cat (G.B.-G.); helena.castane@iispv.cat (H.C.); andrea.jimenez@urv.cat (A.J.); jorge.camps@salutsantjoan.cat (J.C.); jorge.joven@salutsantjoan.cat (J.J.); 3 Department of Geriatric and Palliative Care, Hospital Universitari Sant Joan de Reus, 43204 Tarragona, Spain; gabrielde.febrer@urv.cat (G.D.F.); carlos.vasco@salutsantjoan.cat (C.V.); 4 Department of Pathology, Hospital Universitari Sant Joan de Reus, 43204 Tarragona, Spain; 5 Department of Radiation Oncology, Institut d’Investigacions Mèdiques, Hospital del Mar, Autonomous University of Barcelona, 08193 Barcelona, Spain; 85368@parcdesalutmar.cat; 6 Department of Radiation Oncology, HM Hospitales, 28050 Madrid, Spain; angel.monteroluis@gmail.com; 7 Laboratori de Referència Sud, Hospital Universitari Sant Joan de Reus, 43204 Tarragona, Spain; jmsimo@lrsud.cat (J.M.S.); xgabaldo@lrsud.cat (X.G.-B.); 8 Department of Radiation Oncology, Complejo Hospitalario de Albacete, 02006 Albacete, Spain; ssabaterm@gmail.com

**Keywords:** COVID-19 pneumonia, low-dose radiation therapy, chemokines, paraoxonase-1

## Abstract

The aim of our study was to investigate the changes produced by low-dose radiotherapy (LDRT) in the circulating levels of the antioxidant enzyme paraoxonase-1 (PON1) and inflammatory markers in patients with COVID-19 pneumonia treated with LDRT and their interactions with clinical and radiological changes. Data were collected from the IPACOVID prospective clinical trial (NCT04380818). The study included 30 patients treated with a whole-lung dose of 0.5 Gy. Clinical follow-up, as well as PON1-related variables, cytokines, and radiological parameters were analyzed before LDRT, at 24 h, and 1 week after treatment. Twenty-five patients (83.3%) survived 1 week after LDRT. Respiratory function and radiological images improved in survivors. Twenty-four hours after LDRT, PON1 concentration significantly decreased, while transforming growth factor beta 1 (TGF-β1) increased with respect to baseline. One week after LDRT, patients had increased PON1 activities and lower PON1 and TGF-β1 concentrations compared with 24 h after LDRT, PON1 specific activity increased, lactate dehydrogenase (LDH), and C-reactive protein (CRP) decreased, and CD4+ and CD8+ cells increased after one week. Our results highlight the benefit of LDRT in patients with COVID-19 pneumonia and it might be mediated, at least in part, by an increase in serum PON1 activity at one week and an increase in TGF-β1 concentrations at 24 h.

## 1. Introduction

Coronavirus disease 2019 (COVID-19) continues to spread worldwide at dangerous levels with high morbidity and mortality rates. Although vaccination campaigns are progressing effectively in high-income countries, infection continues to increase in large parts of the world that have poor health infrastructures, and this is a potential source of new variants of SARS-CoV-2. Therefore, medium and long-term efforts aimed at identifying strategies to treat COVID-19, remain peremptory. The treatment of patients who are not candidates for assisted breathing or pharmacological treatments is a clinical challenge of great magnitude. Some patients develop pneumonia that can lead to respiratory failure or acute respiratory distress syndrome. This life-threatening complication is attributed to a hyperinflammatory response that occurs when large numbers of leukocytes (neutrophils, macrophages, and mast cells) become activated and release large amounts of pro-inflammatory cytokines [1]. COVID-19 has been associated with a specific immune profile: a decrease in the cluster of differentiation (CD)3+, CD4+, and CD8+ cells; natural killer cells; and B-cells; a rise in interleukin (IL)-6, IL-4, and tumor necrosis factor alpha (TNF-α); and decrease in IL-10 and interferon (IFN)-γ [2]. In turn, this inflammatory reaction produces oxidative stress. On one hand, this is a defense mechanism against viral infection, but on the other hand, it contributes to aggravating tissue damage. The innate immune system has mechanisms that protect against oxidative stress. Among these mechanisms, the enzyme paraoxonase-1 (PON1) plays an important role [3] and a recent study by our research group has shown that PON1 activity is greatly inhibited in the serum of COVID-19 patients [4].

Several authors have proposed the utility of low-dose radiotherapy (LDRT) in COVID-19 due to its anti-inflammatory effects [5,6,7]. LDRT modulates the function of a variety of inflammatory cells, including endothelial cells, polymorphonuclear leukocytes, and macrophages. The present study aimed to investigate the effects of LDRT in PON1-related variables and cytokines and to analyze their relationship with the clinical and radiological characteristics of patients with COVID-19 pneumonia.

## 2. Materials and Methods

### 2.1. Study Design and Participants

We selected 30 patients from the IPACOVID clinical trial (NCT04380818) included between June and December 2020. This is an ongoing multicenter, prospective, observational study of patients with COVID-19 pneumonia who are not candidates for invasive mechanical ventilation due to their comorbidities, old age and/or general condition, and for whom other therapeutic alternatives are not available. These patients were treated during the acute phase of the viral infection with a single dose of 0.5 Gy radiotherapy to the whole thorax. The protocol details have been published previously [8,9,10]. The study has been approved by the Ethical Institution Research Board of each center and is registered in ClinicalTrials.gov (NCT NCT04380818). All patients read and signed an informed consent prior to inclusion. Serum samples from all participants were obtained immediately before, at 24 h after, and 1 week after LDRT, and stored at –80 °C until analyzed. SaFI ratio [pulse oximetric saturation (SpO_2_)/fraction of inspired oxygen (FiO_2_)] was measured and classified as mild (>300), moderate (201–300), and severe (<200). Other respiratory parameters, such as arterial oxygen partial pressure (PaO_2_) and daily oxygen supply (O_2_, L), were also measured. Radiological findings in lung computed tomography (CT) scans were classified according to the percentage of lung parenchymal infiltrates (>75%, 50–75%, 25–50%, 5–25%, <5%). 

### 2.2. Analytical Measurements

PON1 concentration was analyzed with the Human PON1 ELISA kit from Elabscience^®^ (Houston, TX, USA). PON1 activity was analyzed by the rate of hydrolysis of phenylacetate at 280 nm, in a 9 mM Tris-HCl buffer, pH 8.0, and supplemented with 0.9 mM CaCl2, as previously described [4]. The specific activity of PON1 was calculated as the ratio between PON1 activity and concentration. CCL2, IL-10, IL-4 and INF- were quantified with ABTS ELISA Development kits (Peprotech, London, UK). Transforming growth factor beta 1 (TGF-β1) and TNF-α concentrations were measured by Proteintech ELISA kits (Manchester, United Kingdom). CD4+ and CD8+ cells were quantified by flow citometry (FACSCalibur^TM^ analyzer, Beckton & Dickinson, Franklin Lakes, NJ, USA). D-Dimer was analyzed in a Sysmex CS-2100i^TM^ analyzer (Sysmex GmbH, Norderstedt, Germany). Standard biochemical and hematological analyses were performed in a COBAS^®^ 8000 (Roche Diagnostics, Basel, Switzerland) and a Sysmex XN-1000^TM^ (Sysmex GmbH) automated analyzers. 

### 2.3. Statistical Analysis

The Kolmogorov–Smirnov test was employed to assess the normality distribution of the variables. The Wilcoxon rank-sum test was used to compare quantitative variables, and the Spearman’s Rho test was used to determine correlations between quantitative data. Qualitative variables were analyzed with the χ^2^ test. Statistical significance was set at *p* < 0.05. All the statistical analyses used the Statistical Package for Social Sciences (SPSS 24.0, Chicago, IL, USA), Jupyter notebook from Python, and GraphPad Prism 9 (GraphPad Software, San Diego, CA, USA). The diagnostic accuracy of selected analytical variables was assessed by receiver operating characteristics (ROC) curves and the areas under the curve (AUC) were calculated [11]. 

## 3. Results

### 3.1. Clinical Characteristics of Patients

All the patients included were elderly (mean age 86 years), had several comorbidities, and followed a pharmacological treatment with dexamethasone. At the time of the study, no patient had received any dose of the vaccine of SARS-CoV2. Survival at the end of the study was 83.3%. Table 1 describes the patients’ baseline characteristics. The alterations in respiratory and radiological parameters at baseline, 24 h, and 1 week after LDRT are summarized in Table 2. One week after treatment, we observed a significant increase in SaFI, and a decrease in O_2_ supply. CT scans showed a significant decrease in the degree of lung involvement 1 week after LDRT. No adverse events related to radiation treatment have been observed.

### 3.2. Influence of LDRT in Analytical Variables

**Figure 1:**
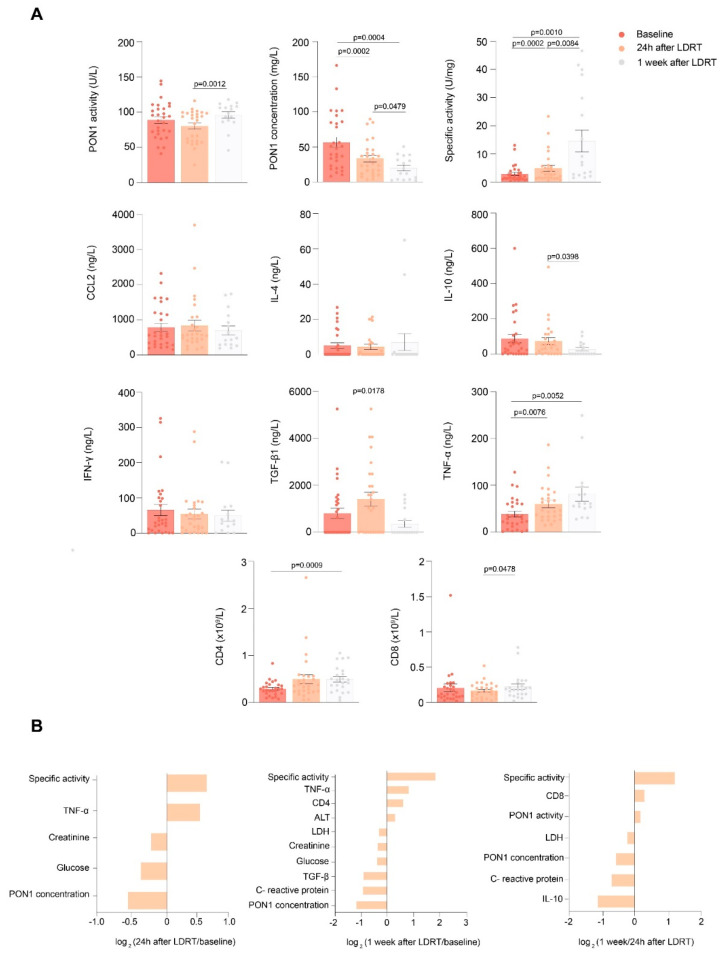
(**A**) Serum paraoxonase-1 (PON1)-related variables and selected cytokines and immunological markers in patients with COVID-19 pneumonia before and after low-dose radiation therapy (LDRT). (**B**) Magnitude of change of selected variables, shown as log_2_fold change. Abbreviations: CCL2, chemokine (C-C motif) ligand 2; CD, cluster of differentiation; IFN-γ, interferón γ; IL, interleukin; LDH, lactate dehydrogenase; PON1, paraoxonase-1; TGF-β1, transforming growth factor beta 1; TNF-α, tumor necrosis factor α.

We observed a significant decrease in serum PON1 concentration, and an increase in TGF-β1 and TNF-α concentrations 24h after LDRT, with respect to baseline. After 1 week, serum PON1 activity and TNF-α concentration increased, while PON1 and TGF-β1 concentrations decreased with respect to 24h. CD4+ and CD8+ lymphocytes increased after 1 week compared to baseline and 24h after LDRT, respectively. PON1 specific activity progressively increased from baseline up to 1 week after LDRT. We did not find any significant changes in CCL2, IL-4, IL-10, and IFN-γ concentrations (Figure 1A). Results of the selected standard biochemical and hematological variables before, at 24 h, and at 1 week after LDRT are shown in Table 3, and the magnitude of change of each significant alteration is expressed as log_2_ fold change (Figure 1B). We observed a significant decrease in lactate dehydrogenase (LDH) activity and C-reactive protein (CRP) concentrations 1 week after LDRT.

**Figure 2:**
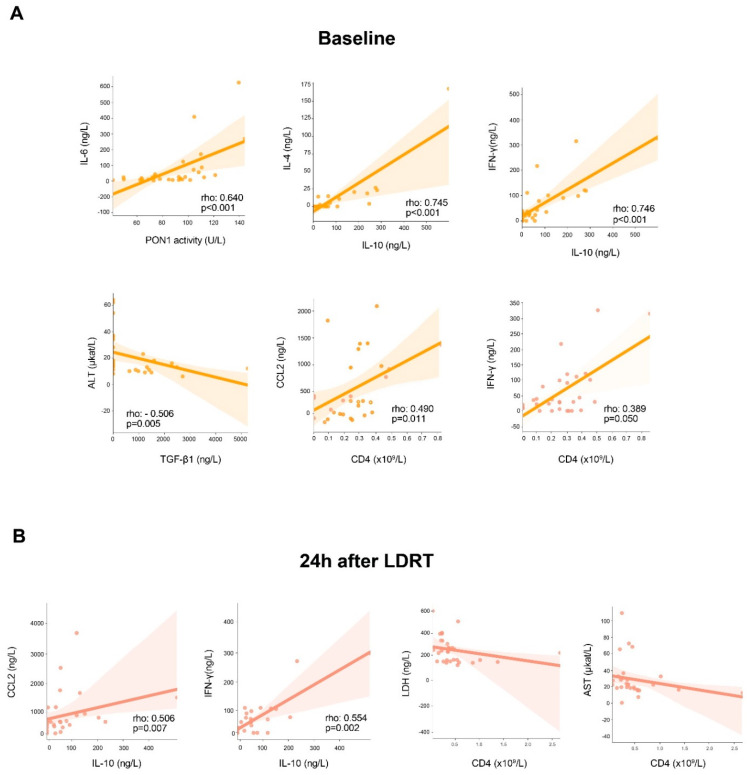
Statistically significant correlations between the analyzed variables at baseline and at 24 h after low-dose radiation therapy (LDRT). Abbreviations: ALT, alanine aminotransferase; CD, cluster of differentiation; IFN-γ, interferon γ; IL, interleukin; LDH, lactate dehydrogenase; PON1, paraoxonase-1; TGF-β1, transforming growth factor beta 1.

Figure 2 summarizes the correlations between all the analyzed parameters. At baseline, we found direct correlations between IL-6 and PON1 activity, IL-4 and IL-10, IFN-γ and IL-10, and CCL2 and CD4+ cells, and an inverse correlation between ALT and TGF-β1 (Figure 2A). Twenty-four hours after LDRT, we found direct correlations between CCL2, IFN-γ and IL-10 concentrations and inverse correlations between LDH, AST, and CD4+ cells (Figure 2B). 

### 3.3. Relationships between Clinical, Radiological and Analytical Variables

**Figure 3:**
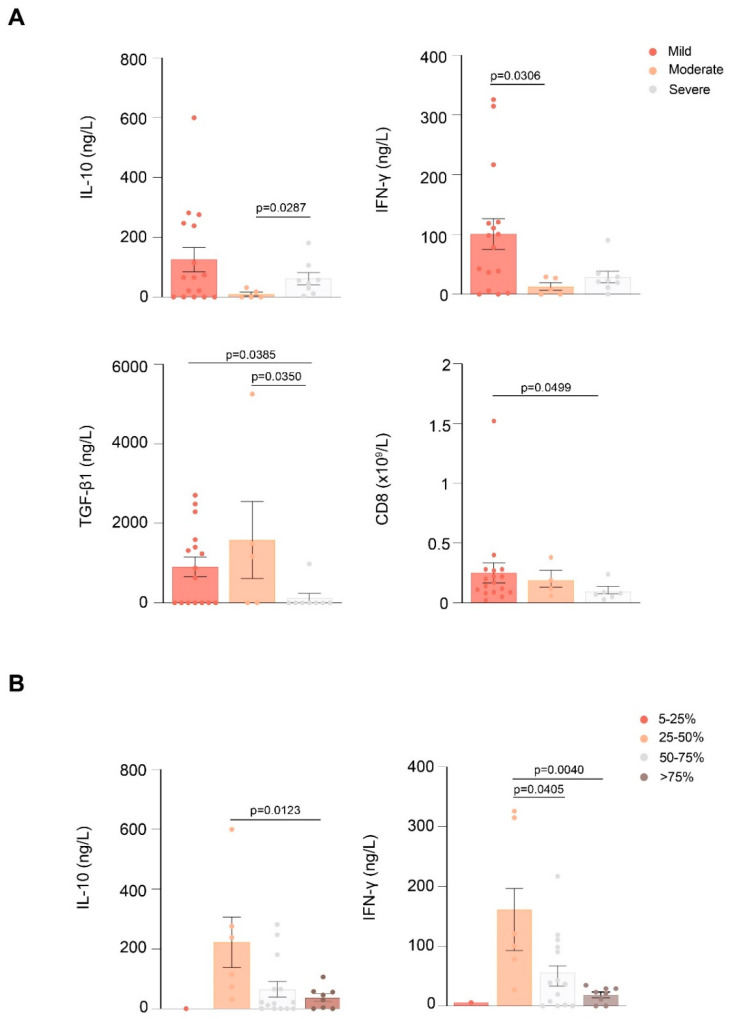
Baseline values of variables showing statistically significant differences between patients with COVID-19 pneumonia according to their SaFI (**A**) or their percentage of lung parenchymal infiltrates (**B**). Abbreviations: CD, cluster of differentiation; IFN-γ, interferon γ; IL, interleukin; TGF-β1, transforming growth factor beta 1.

At baseline, patients with severe respiratory conditions showed lower IL-10, IFN-γ, and TGF-β1 concentrations and a lower number of CD8+ cells than patients with mild and/or moderate respiratory (Figure 3A). Moreover, patients with a higher lung parenchymal involvement measured by CT had lower IL-10 and IFN-γ concentrations than patients with a more preserved lung (Figure 3B). 

**Figure 4:**
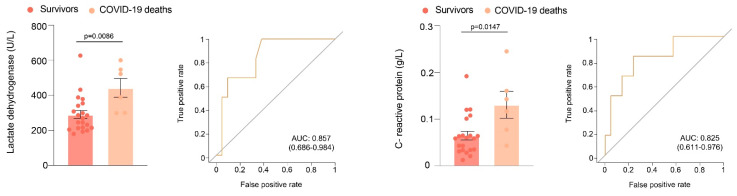
Lactate dehydrogenase (LDH) and C-reactive protein (CRP) levels and receiver operating characteristics plots in patients who died and those who did not.

Baseline LDH activity and C-reactive protein (CRP) concentrations were higher in patients who died from pneumonia than in those who survived. ROC analysis showed AUC’s higher than 0.80 in the discrimination between patients who died and those who did not (Figure 4). 

**Figure 5:**
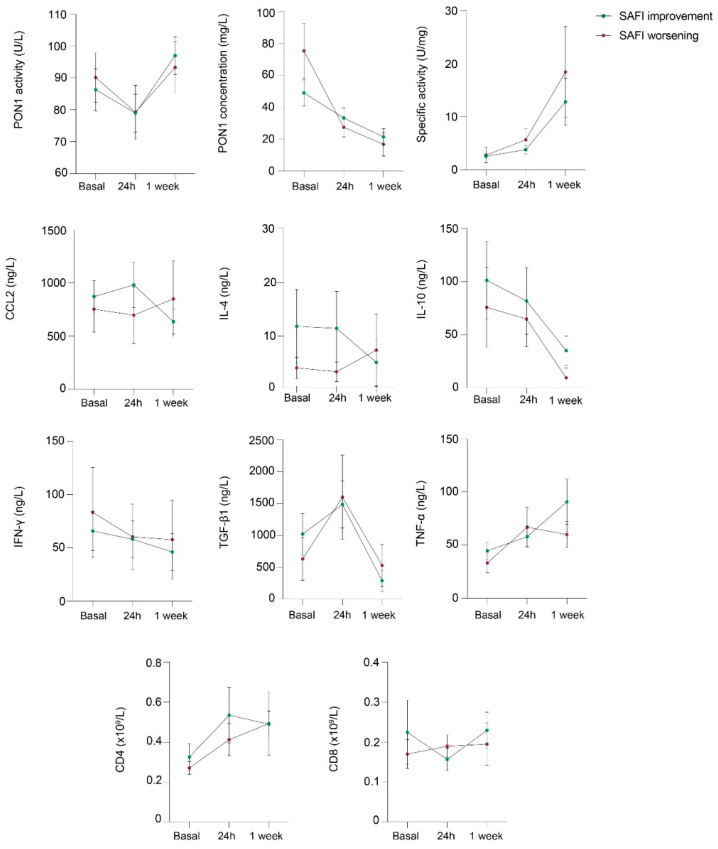
Selected variables in patients with COVID-19 pneumonia according to whether they showed a SaFI improvement or a worsening after 1 week of low-dose radiation therapy. Abbreviations: CCL2, chemokine (C-C motif) ligand 2; CD, cluster of differentiation; IFN-γ, interferon γ; IL, interleukin; PON1, paraoxonase-1; TGF-β1, transforming growth factor beta 1; TNF-α, tumor necrosis factor α.

We did not observe any significant differences in the parameters between the patients who improved in SaFI and those who worsened, although there was a trend towards patients who improved to have higher levels of the anti-inflammatory cytokines IL-4 and IL-10 (Figure 5).

## 4. Discussion

The present study shows that LDRT was associated with a 22.4% average increase in the SaFI ratio one week after treatment in patients who survived, and this was accompanied by a radiological improvement in lung affectation. These results agree with those published previously. Several groups, including our own, have shown that whole-lung radiation at doses of 0.5–1.5 Gy can accelerate the recovery in clinical and radiographic status without acute toxicity [12,13,14,15,16,17,18,19,20,21]. The appearance of new variants and the high degree of vaccination in most high-income countries has changed the scenario since 2020, towards a less severe disease and therefore, the usefulness of LDRT may be lower in the future than in these recent years. This treatment is intended for people with severe lung disease who are not candidates for other treatment options. The question that arises, then, is whether it could be a therapeutic alternative in the future. We think that it is, since the transmissibility of the new variants seems to be higher than that of the previous ones and vaccination rates are still low in many countries of the world, especially among the elderly. This means that, predictably, it will still be possible to find a significant number of severely ill elder patients to whom other treatments cannot be applied and for whom LDRT is still a valid option. One limitation is that LDRT is only available in large or medium-sized hospitals that have a radiotherapy department, being a cost-effective treatment there, and candidate patients should be transferred to centers that have these services.

We now expand these results by delving into some of the possible biochemical mechanisms underlying this effect and investigating the relationships between biochemical parameters and the clinical response. The clinical improvement of these patients was accompanied by a significant increase in PON1 specific activity, mainly related to a decrease in serum PON1 concentration, and anti-inflammatory changes in circulating cytokine concentrations. PON1 is a lactonase and an esterase that degrades lipid peroxides in lipoproteins and cells [3]. In humans, this enzyme is mainly synthesized by the liver, and is found in blood bound to high-density lipoproteins [22,23,24]. The enzymatic action of PON1 is elicited in the circulation within high-density lipoprotein particles but they can also be transported from these particles to the cell membranes [25], especially of epithelial and endothelial cells [26,27,28]. PON1 protein expression is high in lung epithelial cells, since this is one of the epithelia most exposed to oxidative stress or to the aggression of xenobiotics and other external agents [27]. A previous study by our research group showed that serum PON1 activity is decreased, and its concentration is increased in patients with COVID-19 [4]. The present study reports that PON1 activity increases significantly one week after LDRT, while its concentration decreases, the net result being a progressive increase in specific activity (i.e., activity per mg of protein). To understand this, one must take into account that PON1 is a lipoperoxide hydrolase and, to exert its action, the active center of the enzyme has to bind covalently to the substrate molecules, the result being that the enzyme is inactivated [29]. An increase in oxidative stress produces a decrease in serum PON1 activity that occurs, despite an increase in its serum concentration, interpreted by the organism as an attempt to counteract the decrease in enzyme activity. On the other hand, a decrease in the level of oxidative stress would produce changes in the opposite direction: an increase in activity and a decrease in concentration, with a consequent increase in specific activity [30,31,32]. 

In several non-communicable and infectious diseases, changes in PON1 activity are the cause or consequence of alterations in the inflammatory state of the organism. When SARS-CoV-2 infects the lungs, they produce a hyperinflammatory cascade that determines the severity of the infection [1,2]. From this point of view, we have found that patients with SaFI < 300 or lung parenchymal infiltrates > 50% had lower baseline IL-10 and IFN-γ concentrations than patients with milder levels of disease. Our results show that LDRT was associated with a decrease in the circulating levels of some markers of inflammation, such as LDH activity, CRP concentrations, and an increase in the levels of the anti-inflammatory cytokine TGF-β1 at 24 h. The number of CD4+ and CD8+ cells increased. Moreover, patients who respond better to LDRT tended to have higher levels of the anti-inflammatory cytokines IL-4 and IL-10. The effects of LDRT seem to be multifactorial, and reports in non-COVID-19 patients show that they include a decrease in polymorphonuclear cells and endothelial cells, a decrease in the expression of adhesion molecules, a decrease in the production of nitric oxide (NO), an increase the activation of apoptosis mediators, and an increase the production of IL-10 and TGF-β1 [33,34,35,36,37,38]. 

Our findings support that LDRT merits consideration for treating selected patients because it appears to lack acute toxicity in the elderly. It has been reported that low doses of irradiation up to 0.5 Gy are expected to induce a low number of RNA damage events and mutations in the virus, and be of a low selective pressure. A dose of 0.5 Gy in an approximately 30 kb single-stranded virus genome, would produce about 0.005 single-strand breaks per virus (assuming ~1000 strand breaks per ~3 Gb genome) [39]. It is notable that whole-lung LDRT did not induce post-treatment pancytopenia, or immunosuppression, such as glucocorticoids. Therefore, this treatment might be unlikely to worsen whole-body immunity or slow down the clearance of the virus. By contrast, dexamethasone can induce global immunosuppression, which has been to slow down viral clearance in murine models, and remains a concern. In addition, steroids can induce hyperglycaemia, insulin requirements, and superimposed bacterial infections or even sepsis during COVID-19 hospitalization, complicating recovery. The present study suggests that LDRT is an option as a focal anti-inflammatory option without acute effects. This study has several limitations: the number of patients is low because we have limited the requirements for which the treatment would be indicated. In addition, it is not a randomized study, nor does it have a control group, for obvious ethical reasons, since that would mean depriving patients of any therapeutic measure who were at clear risk of death. In addition, although the clinical effect of LDRT on lung function seems to be maintained after one week, our results suggest that the underlying biochemical mechanisms could be partly different from those analyzed, since the concentration of TNF-α increases and those of TGF-β1 and IL-10 decreases after one week, which would indicate a depletion of the anti-inflammatory response, at least through these mediators. This topic deserves further research.

## 5. Conclusions

Despite these limitations, our results highlight the benefit of LDRT in patients with COVID-19 pneumonia and that may be mediated, at least in part, by an increase in serum PON1 activity and specific activity, and a decrease in inflammatory and immunomodulatory markers. As far as we know, this is the first study to provide an insight into the biochemical mechanisms of the effects of LDRT in COVID-19 pneumonia. 

## Figures and Tables

**Table 1:** Clinical variables of patients with COVID-19 treated with low-dose radiation therapy.

Clinical Characteristics	(n = 30)
**Age**	86 (range: 82–88)
**Sex**	
Female	15 (50%)
**Comorbidities**	
Neurologic diseases	8 (26.7%)
Cardiovascular diseases	26 (86.7%)
Respiratory diseases	9 (30%)
Other comorbidities	27 (90%)
COVID-19 symptoms (days)	5 (range: 4–7)
**Pharmacological treatment**	
Corticoids (dexametasone)	30 (100%)
Remdesivir	-
Tocelizumab	-
**Final status**	
Survival	25 (83.3%)
Death from COVID-19	5 (16.7%)

Results are shown as frequencies (%) or medians (interquartile range).

**Table 2:** Respiratory and radiological parameters at baseline, 24h, and 1 week after low-dose radiation therapy (LDRT).

	Baseline n = 30	24 h after LDRT n = 28 *	1 Week after LDRT n = 23 *	*p* Value ^1^	*p* Value ^2^	*p* Value ^3^
**Respiratory parameters**						
SaFI ratio	330.5 (181.7–350)	346 (216.2–350)	404 (343–462)	0.043	0.001	0.004
PaO_2_/FiO_2_	313 (212.5–333)	332 (254–336)	336 (270–381)	0.215	0.035	0.256
FiO_2_ (%)	28 (28–50)	28 (28–46.2)	21 (21–28)	0.035	0.002	0.020
O_2_ (L)	3 (2.7–10.5)	3 (2–9)	2 (0–4)	0.016	0.002	0.006
O_2_ saturation (%)	95 (91–97)	96 (94–98)	97 (96–98)	0.119	0.001	0.258
Respiratory rate (brpm)	18 (17–19)	16 (16–17)	16 (15–17)	0.006	<0.001	0.071
**Radiological parameters**						
CT lung involvement (%)						
<5	-	-	3 (10)	-	0.163	-
5–25	1 (3.3)	-	12 (40)	-	<0.001	-
26–50	6 (20)	-	4 (13.3)	-	0.969	-
51–75	14 (46.7)	-	4 (13.3)	-	0.042	-
>75	9 (30)	-	-	-	0.010	-

* Values of SaFI ratio from two patients were not available and therefore they were excluded from the analysis. Results are shown as frequencies (%) or medians (interquartile range). *p* value ^1^: 24 h after LDRT with respect to baseline; *p* value ^2^: 1 week after LDRT with respect to baseline; *p* value ^3^: 1 week after LDRT with respect to 24 h after LDRT. Abbreviations: SaFI, Pulse oximetric saturation (SpO_2_)/fraction of inspired oxygen (FiO_2_); PaO_2,_ oxygen partial pressure; FiO_2,_ fractional inspired oxygen; CT, computed tomography.

**Table 3:** Biochemical parameters at basal, 24h and 1 week after low-dose radiation therapy (LD-RT).

	Baseline n = 30	24h after LDRT n = 28 *	1 Week after LDRT n=23 *	*p* Value ^1^	*p* Value ^2^	*p* Value ^3^
**Glucose, mmol/L**	7.2 (6.2–10.5)	5.8 (4.8–7.5)	5.4 (4.7–7.1)	0.002	0.011	0.553
**Creatinine, µmol/L**	94 (75–165.7)	73.4 (48.6–130.8)	87.5 (62.8–107)	<0.001	0.005	0.972
**Lactate dehydrogenase, U/L**	299 (216.5–388.2)	282.5 (201–371.2)	218.5 (172.5–285.5)	0.127	0.002	0.007
**Hemoglobin** **, g/dL**	11.8 (10.7–12.5)	11 (10–12.2)	11.2 (10.1–12.1)	0.086	0.074	0.322
**Ferritin, ng/mL**	574 (285.2–1466.7)	627 (315–2599.7)	493 (228.7–1012.7)	0.607	0.231	0.093
**AST** **, µkat/L**	26 (18–38.5)	22 (16.2–31.5)	19 (15–28)	0.721	0.465	0.795
**ALT** **, µkat/L**	14.5 (11–24.2)	17.5 (13.2–24.2)	21 (17–37)	0.089	0.041	0.086
**C-reactive protein, mg/dL**	6.2 (3.3–11.7)	5.2 (2.5–8)	3.4 (0.3–5.9)	0.054	0.001	0.013
**Leukocytes, x10^9^L**	7.6 (4.4–13.6)	7.3 (4–11)	7.8 (5.6–11.4)	0.050	0.465	0.277
**Lymphocytes, x10^9^L**	0.8 (0.5–1.2)	0.9 (0.6–1)	1 (0.5–1.8)	0.682	0.369	0.058
**Platelets, x10^9^L**	195.5 (162–253.7)	219 (141.2–253)	234 (142–331)	0.764	0.107	0.144
**D-Dimer,** **μ** **g/L**	1020 (755–1965)	1070 (770–2220)	1060 (660–2700)	0.767	0.964	0.796
**CD4+ cells, x10^9^/L**	0.3 (0.2–0.4)	0.3 (0.2–0.5)	0.5 (0.2–0.7)	0.067	0.002	0.191
**CD8+ cells, x10^9^/L**	0.1 (0.08–0.2)	0.1 (0.08–0.2)	0.2 (0.1–0.3)	0.389	0.088	0.047
**IL-6, ng/L**	24 (9.5–64)	36.5 (9.2–72.5)	17 (5–29)	0.307	0.114	0.145

* Values of SaFI ratio from two patients were not available and therefore they were excluded from the analysis. Results are shown as medians (interquartile range). *p* value ^1^: 24h after LDRT with respect to baseline; *p* value ^2^: 1 week after LDRT with respect to baseline; *p* value ^3^: 1 week after LDRT with respect to 24 h after LDRT. Abbreviations: AST, aspartate transaminase; ALT, alanine transaminase; CD4, cluster of differentiation 4; CD8, cluster of differentiation 8; IL-6, interleukin-6; LDRT, low-dose radiation therapy.

## Data Availability

Data is contained within the article.
